# Bile Acid Synthesis: From Nature to the Chemical Modification and Synthesis and Their Applications as Drugs and Nutrients

**DOI:** 10.3389/fphar.2018.00939

**Published:** 2018-09-25

**Authors:** Tanja M. Šarenac, Momir Mikov

**Affiliations:** ^1^Faculty of Medicine, University of Novi Sad, Novi Sad, Serbia; ^2^Department of Pharmacology, Toxicology and Clinical Pharmacology, University of Novi Sad, Novi Sad, Serbia

**Keywords:** BAs, chemical modification, drugs, biosynthesis of BAs, chemical synthesis

## Abstract

Bile acids (BAs) are amphiphilic molecules with 24 carbon atoms and consist of a hydrophobic and a rigid steroid nucleus, to which are attached a hydrophilic hydroxyl group and a flexible acidic aliphatic side chain. The steroidal core of BAs constitutes a saturated cyclopentanoperhydrophenanthrene skeleton, consisting of three six-membered (A, B, and C) and one five-membered ring (D). Primary BAs are produced in the hepatocytes, while secondary BAs are formed by modifying the primary BAs in the intestinal lumen, i.e., by the reactions of 7α-dehydroxylation and deconjugation of cholic acid (CA) and chenodeoxycholic acid (CDCA). The most important secondary BAs are deoxycholic acid (DCA) and lithocholic acid (LCA). The BAs realize their effects through nuclear farnesoid X receptors (FXRs) and membrane TGR5 receptors. It has been found that BAs are also associated with other receptors such as the vitamin D receptor (VDR), from which the most significant ligand is calcitriol, as well as with pregnane X receptor (PXR) and potentially with the constitutive androstane receptor (CAR), whose ligands are numerous, structurally different xenobiotics that show greater affinity to BAs. The BAs as therapeutic agents (drugs) have the potential to produce beneficial effects in cases of sexually transmitted diseases, primary biliary cirrhosis (PBC), primary sclerosing cholangitis, gallstones, digestive tract diseases, cystic fibrosis, and cancer. Ursodeoxycholic acid (UDCA) was the only drug approved by the US Food and Drug Administration (FDA) for the treatment of PBC. In this paper, the different pathways of bile acid biosynthesis are explained as well as chemical modifications and the synthesis of different keto derivatives of BAs. Also, the effects of BAs on digestion of nutrients, their role as drugs, and, in particular, the emphasis on the hypoglycemic properties of 7α, 12α-dihydroxy−12–keto−5β-cholanic acid in the treatment of diabetes mellitus are examined in detail.

## Introduction

Bile acids are classified into a heterogeneous group of amphiphilic steroidal molecules. They consist of a saturated tetracyclic hydrocarbon cyclopentanoperhydrophenanthrene ring, containing three six-membered rings (A, B, and C) and a five-membered ring (D). Also, BAs have a rigid steroid nucleus and a short aliphatic side chain. There are angular methyl groups at positions C-18 and C-19 (Kuhajda et al., 2006). In higher vertebrates, the bile acid nucleus is curved because the A and the B rings are in a *cis*-fused configuration. In addition, there are angular methyl groups at positions C-18 and C-19. The BAs in lower vertebrates are known as allo-BAs. In this case, A i B rings are trans linked (5α-stereochemistry). There are four different types of BAs (C_27_, C_26_, C_25_ and C_24_), and they occur in the less -developed forms of life. There are two major classes of BAs depending on the length of the side chain: C_27_ and C_24_ BAs (Kuhajda et al., 2006). In higher vertebrates, C_24_ BAs constitute a major part of the bile. The BAs are facially amphipathic, i.e. they contain both hydrophobic (lipid soluble) and hydrophilic (polar) faces. Iida and coworkers explained the chemical synthesis of several rare BAs. An unusual bile acid, 16α-chenodeoxycholic acid (CDCA), has recently been isolated from certain species of storks and herons (Pellicciari et al., 2016). This bile acid was named avicholic acid. It was formed from CDCA. This rare bile acid is prepared from readily available CDCA using Breslow's biomimetic remote functionalization in a key step. The BAs are conjugated to glycine or taurine to yield the conjugated form of BAs. They are synthesized in the hepatocytes as the main product of cholesterol catabolism (Björkhem et al., 2010). Primary BAs are produced in the hepatocytes (Mukhopadhyay and Maitra, 2004a). By modifying primary BAs in the intestinal ileum, secondary BAs are formed. Due to their amphiphilic structure, BAs allow the emulsification, digestion, and absorption of liphophilic xenobiotics after a meal. Also, solubilization of cholesterol in the bile and biliary secretion of phospholipids take place, which have been the main roles considered over decades. Mikov et al. (2007) The BAs also have significant antibacterial properties, influencing the composition of the intestinal microflora and maintaining the sterility of the biliary tract. They exhibit a cytotoxic and membranolytic effect due to their detergent activity at the cell membrane level in concentrations above the critical micelle concentration (CMC). Bile acids are surface active molecules, which are characterized by a tendency for the spontaneous formation of aggregates on the boundary surface of the hydrophilic and lipophilic faces, as long as they are present in concentrations above the CMC. They have the ability to aggregate into micelles. Hydroxyl groups at positions C_3_, C_7_, and C_12_, which are evolutionarily highly conserved in higher vertebrates, represent an optimal configuration for the establishment of hydrogen bonds (Natalini et al., 2014). Bile acids with hydroxyl groups localized on both sides of the hydrophobic steroid core (α- and β- orientation) are more hydrophilic than the molecules with the same number of hydroxyl groups only in the α-orientation. Thus, they have less ability of aggregat, i.e., possess a higher CMC value, due to their hydrophilicity. The significance of the contact of hydrophobic surfaces in bile acid aggregation is evident in the differences between the CMC values of the two epimers of BAs (chenodeoxycholic acid, 3α,7α-dihydroxy−5β-cholan−24–oic acid, CDCA) and ursodeoxycholic acid (3α,7β-dihydroxy−5β-cholan−24–oic acid, UDCA). Hydrophilicity of free and conjugated BAs decreases in the following order–UDCA >CA > CDCA > DCA > LCA; taurine salts of BAs > glycine salts of BAs > free bile acid (Mikov et al., 2007). The structure of the side chain influences the CMC value by reducing the number of carbon atoms, thus increasing the ability to associate as micelles (Natalini et al., 2014). The formation of micelles is primarily due to the hydrophobic interactions of nonpolar convex β surfaces of the nucleus and hydrogen bonds established through polar structures. Bile salt mixed micelles are promising systems for drug delivery, and they can solubilize cholesterol, lecithin, and monoglycerides, which are intrinsically water insoluble. (Natalini et al., 2014) The aqueous solubility of cholesterol (~1 nM) can increase more than a million fold in the presence of bile-salt micelles. Hydrosolubility is primarily determined by substituents on the steroid core, especially when the hydroxyl groups are localized from the same, concave, α-surface of a steroid nucleus, the hydrosolubility increases due to the cooperative formation of hydrogen bonds of the hydroxyl groups with the solvent. In contrast, the β-stereoisomerization of hydroxyl groups reduces the potential of amphiphilic hydrophobic associations. In concentrations above the CMC, BAs form the small so-called primary micelles (2–10 aggregation units), while at higher concentrations, secondary micelles are formed by the aggregation of primary ones (Poša et al., 2014). The size and shape of the aggregates, in addition to the structure of BAs, are influenced by the conditions of the environmental factors such as pH, temperature, and ionic strength of the solution. Addition of electrolytes reduces the repulsive electrostatic interactions between charged groups, reducing CMC and promoting the formation of micelles. Lowering the pH to the value close to the pKa of BAs leads to the partial protonation of bile anhydride by increasing the CMC. The pKa values of BAs are significantly higher in micellar aggregates compared with the monomer form due to the electrostatic effect (Natalini et al., 2014). They are characterized by antimicrobial activity and prevent excessive development of bacteria in the small intestine. They act as signaling molecules that regulate their own synthesis through activation of nuclear receptors (FXR) and membrane TGR5 receptors and modulate metabolic pathways involving lipoproteins, glucose and drugs. Bile acids have the ability to facilitate the transport of molecules through biomembranes and improve the pharmacokinetic properties of drugs, whose absorption being incomplete and if they have low bioavailability after oral administration (Mikov et al., 2007; Stojančević et al., 2013). The number and position of the hydroxyl groups of BAs directly determine the physico–chemical properties and emulsification potential and the formation of micelles as described by the value of CMC (a higher value of CMC indicates a lower potential for the formation of micelles). In this context, the determination of the CMC value and the resulting cytotoxic potential are essential steps in the characterization of the molecules. Numerous studies have contributed to the understanding of how specific chemical modifications of the steroid core and the side chain of BAs induce different conformational changes that then alter the physico–chemical properties, metabolic properties, distribution in different body compartments and tissues and the cyto(toxic) profile of new analogs. The BAs have more recently been found to act as signaling molecules, notably through the farnesoid X receptor (FXR), a nuclear receptor expressed in the liver, intestine, adrenal glands, and kidneys that has a central role in the synthesis and enterohepatic circulation of BAs. Understanding the relationship between the structure and physico–chemical and physiological properties is a key factor that can contribute to the identification of new bile acid derivatives with favorable pharmacodynamic and pharmacokinetic characteristics as the candidate molecules for testing in preclinical and clinical studies (Chiang, 2009).

Synthesis of BAs represents the dominant metabolic pathway of the catabolism of cholesterol (Björkhem et al., 2010). The conversion of cholesterol to BAs involves a multiplicative enzymatic process, wherein the hepatocytes contain an entire set of 17 enzymes necessary for modifying the cholesterol steroid core, removing the side chain, and conjugation with glycine (~75%) and taurine (25%), resulting in primary BAs–(3α, 7α, 12α-trihydroxy-5β-cholan-24-oic acid (cholic acid, CA) and 3α, 7α-dihydroxy-5β-cholan-24-oic acid (CDCA) (Li et al., 2013). The conversion of cholesterol to BAs involves the processes of hydroxylation, saturation of the double bond between the C5–C6, the epimerization of the C3 hydroxyl group, and the oxidative removal of the three carbon units from the side chain. Biosynthetic reactions take place in the endoplasmic reticulum, mitochondria, cytoplasm, and peroxisomes. Biosynthesis of BAs takes place through four different pathways: classic, alternative, Yamasaki, and 25–hydroxylation pathways. The exact sequence of the biosynthetic step has still not been defined since many intermediates are substituents for the same enzymes. Also, the transport of BAs and intermediates between different subcellular compartments and the course of biosynthesis are still not well known (Li et al., 2013).

### The classic pathway of biosynthesis of BAs

The classic or neutral pathway of the bile acid synthesis cascade is the most important biosynthetic mechanism responsible for the production of 90% of the total amount of BAs. This way, cholic acid (CA) and CDCA are synthesized in almost equal amounts. The 7α-hydroxylase cholesterol (CYP 7A1) is a key enzyme of this catabolic pathway that determines the size of the bile acid pool, catalyzing the hydroxylation of cholesterol to 7α-hydroxycholesterol (Björkhem et al., 2010). Modification of the steroid ring precedes the oxidative shortening of the aliphatic side chain. The 3β-hydroxy–Δ^5^-C_27_-oxidoreductase (HSD3B7) converts 7α-hydroxycholesterol to the 3–oxo–Δ^4^-form after which Δ^4^-3–oxosteroid−5β-reductase (AKR1D1) reduces the Δ^4^ double bond leading to the formation of 5β-hydrogen configuration (Russell, 2003).

The final step in modifying the ring structure is the reduction of the 3–oxo group into the 3α-hydroxyl group by the 3α-hydroxysteroid dehydrogenase (AKR1C4) enzyme. When the hydroxylation reaction takes place in the C_12_ position with sterol, 12α-hydroxylase (CYP 8B1) produces a CA; however, if there is no hydroxylation in this position, it produces a CDCA (Li et al., 2013). Activity of the CYP8B1 enzyme determines the overall hydrophobicity of the pool of BAs, since CA is more hydrophilic compared with CDC. After modification of a ring structure, the carboxyl group is formed at the C_27_ position by the mitochondrial sterol 27–hydroxylase (CYP 27A1), forming C_27_ bile intermediates: 3α, 7α-dihydroxy−5β-cholestanoic acid (DHCA) and 3α, 7α, 12α-trihydroxy−5β-cholestanoic acid (THCA). The C_27_ bile intermediates are then activated into the corresponding coenzyme A (CoA) ester using two enzymes located in the endoplasmic reticulum: bile acid–CoA synthetase (BACS) and a very long chain acyl–CoA synthetase. Then, activated CoA C_27_ bile esters are transported in peroxisome membrane protein using peroxisomal membrane protein 70 (PMP 70, ABCD3), where prior to trimming the side chain, chiral carbon C_25_ racemizes from the *R–* to *S–* configuration using α-methylacyl CoA racemase (AMACR), after which the side chain can be shortened by peroxisomal β-oxidation. In the peroxisomes, (D / T) HC–CoA is oxidized by acyl CoA oxidase 2 (ACOX_2_), forming a double bond at position C_24_ (Chiang, 2015). This double bond is hydroxylated into 24–hydroxy–(D / T) HC–CoA followed by the dehydrogenation into the 24–keto (D / T) HC–CoA, and D–bifunctional protein (DBP) catalyzes both reactions (DBP). Propionyl CoA, CDCA–CoA, or CA–CoA are produced by the thiolytic separation of the resulting ketone using a sterol carrier protein X (SCPx) (Li et al., 2013).

### The alternative pathway of biosynthesis of BAs

**Scheme 1:**
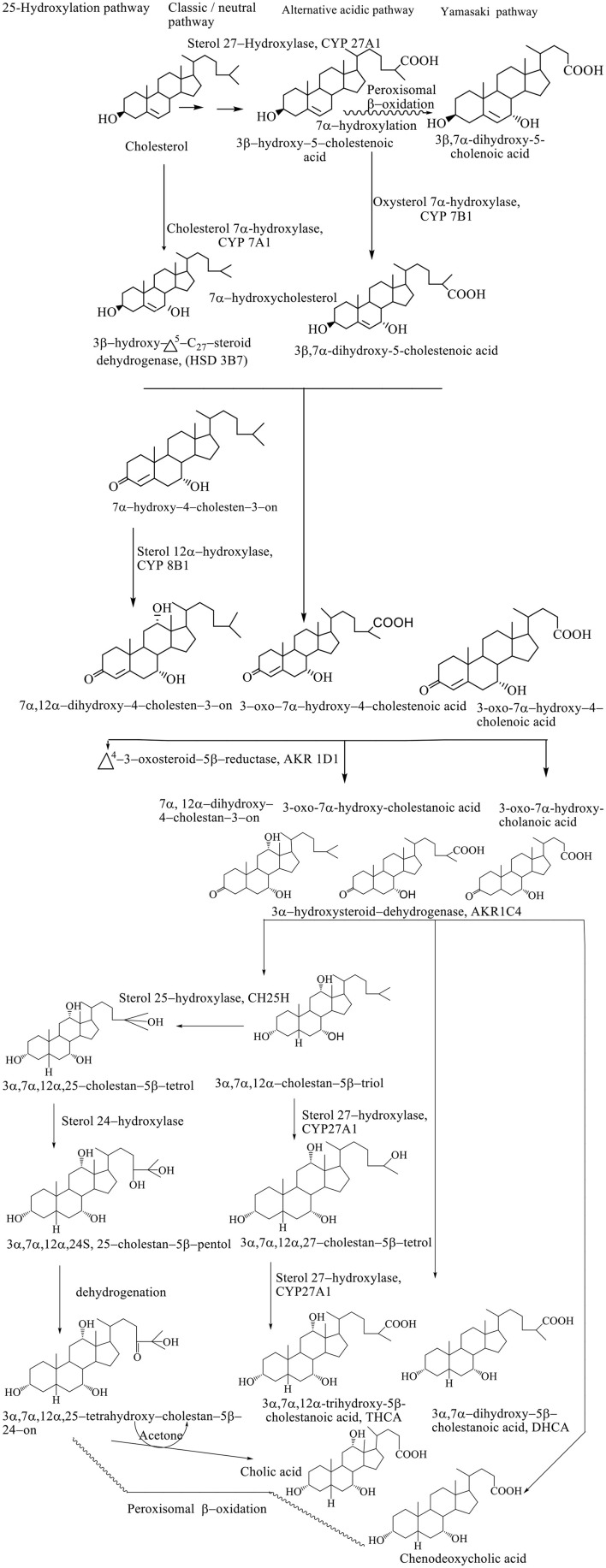
Pathways of biosynthesis of BAs (Kevresan et al., 2007).

An alternative pathway (also known as the acidic pathway due to the synthesis of acidic intermediates) involves the conversion of C_27_ BAs as well as oxysterols formed in different cell types, which are then transported to the liver and metabolized to BAs. Mitochondrial CYP 27A1 and microsomal oxysterol 7α-hydroxylase are key enzymes that make it possible to shorten the side chain in C_24_ BAs and 7α-hydroxylation in the hepatocytes with the highest percentage of CDCA (Kevresan et al., 2007). Alternatively, less than 10% of the total amount of BAs is synthesized. The alternative pathway is thought to be significantly more active in childhood, while later on in the course of life, the classical pathway becomes significant in the contribution to the composition of bile acid pool (Scheme 1; Sarenac and Mikov, 2017).

### The yamasaki pathway of biosynthesis of BAs

In the Yamasaki pathway, the first reaction of the biosynthesis of BAs takes place similar to the alternative pathway resulting in the formation of C_24_ BAs and 3β-hydroxy−5–cholenoic, followed by structural modifications of the steroid ring. In humans, 7α-hydroxylation before or after peroxisomal β-oxidation results in the formation of 3β, 7α-dihydroxy−5–cholanoic acid, a CDCA precursor as the most important product in the biosynthetic pathway. The presence of monohydroxy BAs in the fetal bile and relatively high concentrations of these BAs in meconium and amniotic fluid suggest the importance of this pathway during development (Scheme 1; Kevresan et al., 2007).

### 25–hydroxylation pathway of bile acid biosynthesis

Synthesis of C_24_ BAs after structural modification of the ring, without the need for 27–hydroxylation and consequent β-oxidation implies a 25–hydroxylation pathway of biosynthesis. Microsomal 25–hydroxylase (CH25H) catalyzes for formation of 3α, 7α, 12α-trihydroxy−5β-cholestane−25–tetrol, which is further transferred to 24S–pentol by hydroxylation, followed by dehydroxylation, which produces the 24–oxo–tetrol, which is degraded to CA and acetone (Scheme 1; Kevresan et al., 2007).

The BAs are not excreted in the bile ducts as free carboxylic acids, but, previously, the carboxyl group is activated with acetyl CoA, and then the resulting ester is linked by an amide linkage with the amino acids, glycine or taurine. This process is catalyzed by the enzyme (BACS) and bile acid–CoA: amino acid *N* acetyl transferase. The amide link increases the ionization constant of BAs (pKa~ 5), so that the conjugates of glycine have a pKa value of about 3 (at concentrations below CMC), while the presence of the sulphuric group of taurine provides pKa < 2(Sarenac and Mikov, 2017). By the conjugation process, there is a possibility of reducing the precipitation of bile salts, as well as increasing hydrophilicity of molecules with consequently reduced potential for passing through the cell membrane and reduced cytotoxic and membranolytic properties (Kevresan et al., 2007). Free BAs can diffuse through the cell membrane, while conjugates with glycine or taurine and bile salts are transmitted by active transport using ATP–binding cassette type protein (ATP-binding cassette, ABC). The secretion of bile salts from the hepatocytes into the lumen of the biliary canaliculus is mediated by two ABC transporters: the pump for the export of bile salts, (Bile Salts Export Pump (BSEP), the ATP–binding cassette of subfamily B article 11 (ABCB11), encoding *ABCB11* gene) and the multi associated protein–2, MRP2 (Multidrug Resistance–Associated Protein–2 and ATP–linking cassette of subfamily C article 2 (ABCC2), encoding the *ABCC2* gene), which are the basic mechanisms for the secretion of the bile. Mutation of BSEP encoding genes are responsible for the development of progressive familiar intrahepatic type 2 cholestasis and accumulation of toxic BAs in hepatocytes, while mutations in MRP2 are basis of Dubin–Johnson syndrome (Mikov et al., 2007).

## Intestinal phase of biotransformation of BAs

After ingestion of a fat–containing meal, BAs released from the gall bladder into the duodenum and the intestinal lumen participate in the formation of mixed micelles containing cholesterol, phospholipids, and bile salts intercalated between the polar heads of phospholipids.

Formation of micelles facilitates the digestion and absorption of lipids and liposoluble vitamins from food and aids in the action of pancreatic enzymes. Intestinal microflora has an extremely significant effect on the metabolism of BAs with the primary aim of reducing their bacterial activity. In the lumen of ileum and colon, conjugated BAs are subjected to the deconjugation process under the influence of bacterial hydrolases. From the reaction of 7α-hydroxylation is formed a 3α,12α-dihydroxy−5β-cholan−24–oic acid, while 7α-dehydroxylation of CDCA produces 3α-hydroxy−5β-cholan−24–oic acid, i.e. lithocholic acid (LCA). Other changes in the structure of BAs include oxidation of hydroxyl groups into oxo groups, epimerization of C_3_, C_7_ or C_12_ hydroxyl groups and isomerization of the compound between rings A / B (Sarenac and Mikov, 2017). It results in the epimerization of the 7α-hydroxyl group of CDCA to the 7β-epimer of UDCA with properties favorable to both the host organ and the intestinal bacteria (Mikov et al., 2007) (Scheme 1).

## Enterohepatic recirculation and excretion of BAs

Homeostasis of BAs in enterohepatic circulation is controlled by genes of nuclear receptors (NR_s_). Apart from the nuclear receptors as intracellular bile acid sensors, some cells also contain bile acid receptors at the cell surface including a G–protein coupled receptor (TGR 5 / M–BAR / GPBAR1). These regulatory networks under physiological conditions preserve the enterohepatic circulation of BAs and limit the intracellular levels of potentially toxic BAs. Deconjugated BAs are reabsorbed passively (Roberts et al., 2002).

**Figure 1:**
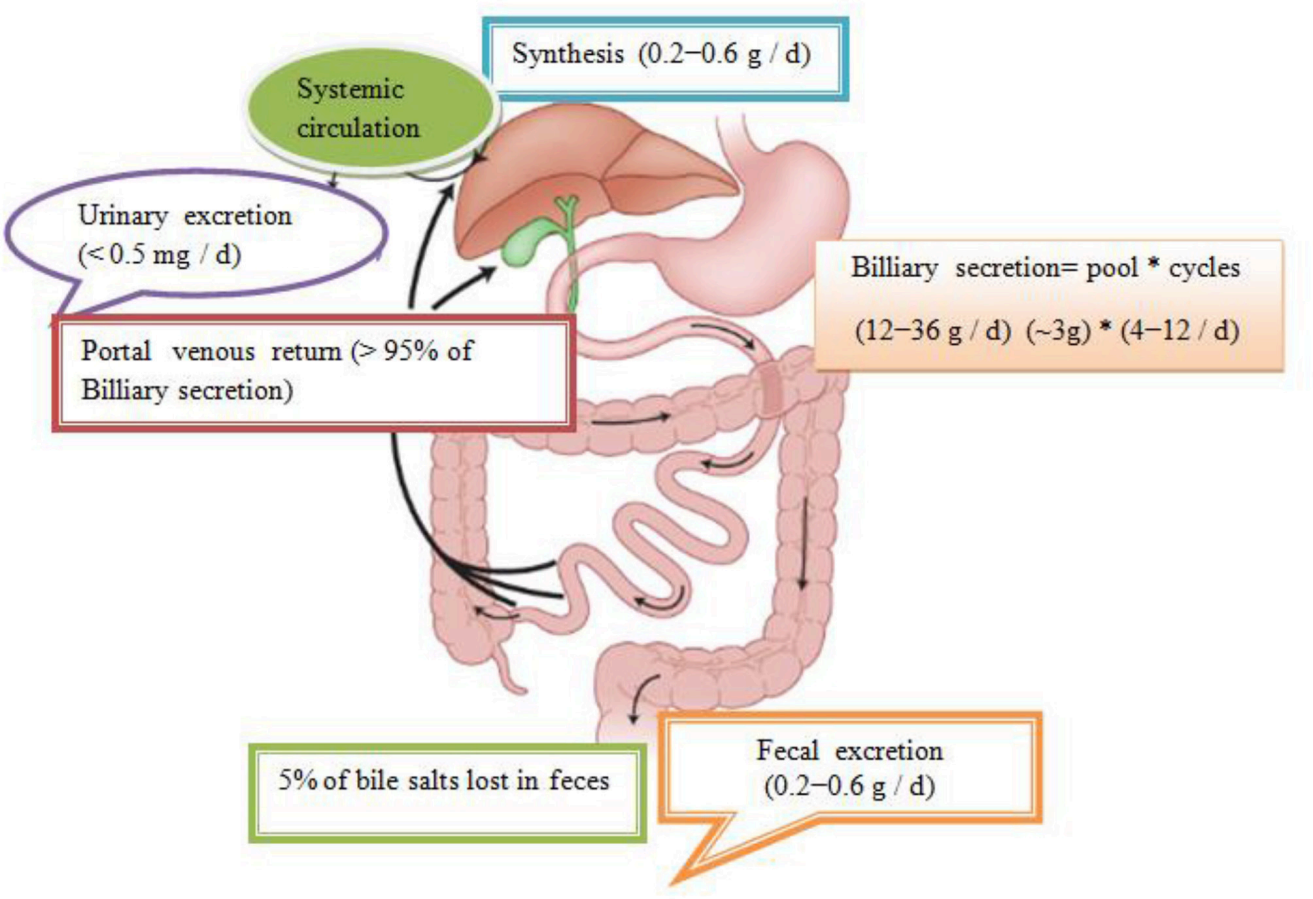
Enterohepatic recirculation of bile salts. Bile salts entering the intestinal tract may be absorbed into the portal circulation where they can be removed from systemic circulation by hepatic uptake. The compound may then be excreted into the bile and pass back into the intestinal tract and become available for enterohepatic cycling. Biotransformation in enterocytes, hepatocytes, and the intestinal tract and throughout the body can convert the drug into metabolites, which may undergo enterohepatic cycling or escape into the urine and feces. Some very lipophilic solutes may bypass the portal circulation and be absorbed into the systemic circulation via the lymphatic system (Mikov et al., 2007).

In total, about 95% is effectively resorbed at the level of the distal ileum by the apical Na–dependent bile acid transporter, (ASBT) encoding the *SLC 10A2* gene), localized in the apical membrane of the enterocyte (Roberts et al., 2002). The intestinal Bile Acid–Binding Protein (IBAB–P), gastrotropin, encoded by the *FABP6* gene provides the trans–enterocytic transport of BAs, while on the basolateral pole of ileal enterocytes, heterodimeric Organic Solute Transporter α / β (OSTα / β) provides their efflux in the portal circulation. Bile salts linked to albumin are transported to hepatocytes, which are taken over by means of Na–dependent cotransporter of BAs, (Sodium Dependent Bile Acid Transporter (NTCP), which encodes the *SLC10A1* gene), while unconjugated BAs are transmitted using Na^+^ independent multispecific transporter of organic anions (Organic Anion Transporter (OATP_s_) and SLC 21A transport proteins) localized in the membrane of the sinusoidal hepatocyte pole. The composition of the pool of BAs (conjugated after release from cholecysts and deconjugated under the action of intestinal microflora in intestinal lumen depends on the availability of nutrients, i.e. the state of satiety and hunger and consists approximately 30% CA 40% CDCA, 20–30% deoxycholic acid (DCA), and less of 5% LCA (Roberts et al., 2002). The LCA as a high toxic bile acid is mostly excreted by feces. A small amount of LCA, which is recycled back into the liver, before repeated rebilayer secretion is subjected to sulfoconjugation at the 3–hydroxy position of sulfotransferase 2A1 (SULT2A1). Sulfoconjugated BAs are almost not reabsorbed by the most important transport proteins, and they are excreted from the body. In kidney glomerulus, BAs are filtered into the primary urine after which they are almost completely reabsorbed with ASBT transporter in the membrane of proximal renal tubulocytes. The pool of BAs (total amount of about 1.5–4 g) is recycled from 4 to 14 times during the day. The fraction that is excreted by the feces per cycle (about 5%, i.e., 0.2–0.6 g / day) is compensated by the synthesis from cholesterol–a mechanism that significantly regulates the plasma cholesterol concentration. Daily amount of newly synthesized BAs in the organism of an adult is about 500 mg, which represented about 50% of cholesterol turnover (Figure 1; Mikov et al., 2007).

## Oxidation reaction of BAs

**Scheme 2:**
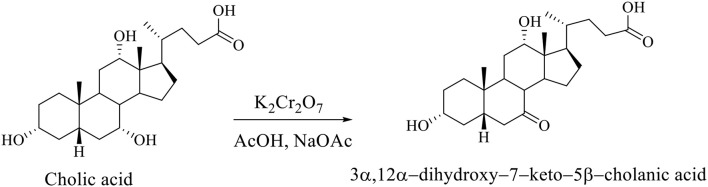
Regioselective oxidation of the CA to 3α,12α-dihydroxy−7–keto−5β-cholanic acid (Kuhajda et al., 2007).

Regioselective oxidation of hydroxyl group at C_7_ of CA was carried out with an aqueous solution of potassium dichromate in acetic acid in the presence of sodium acetate to 3α,12α-dihydroxy−7–keto−5β-cholanic acid (Scheme 2; Kuhajda et al., 2007).

**Scheme 3:**
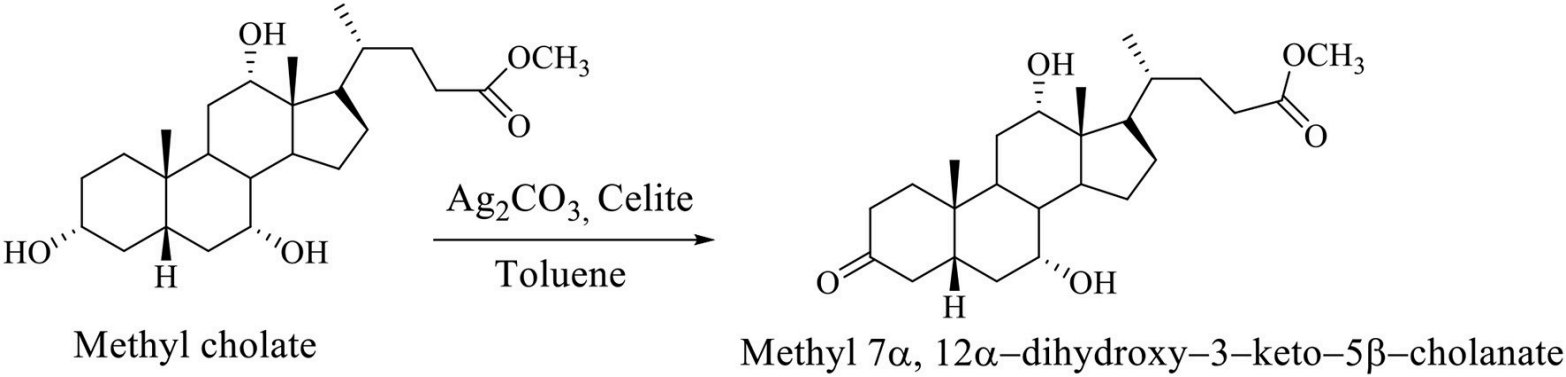
The selective oxidation of methyl cholate to the methyl 7α,12α-dihydroxy−3–keto−5β-cholanate (Kuhajda et al., 2007).

The selective oxidation of the OH group at C_3_ carbon of the methyl cholate is carried out on Celite, which is impregnated with Ag_2_CO_3_ in boiling Toluene, whereby methyl 7α,12α-dihydroxy−3–keto−5β-cholanate is formed (Scheme 3; Kuhajda et al., 2007).

**Scheme 4:**
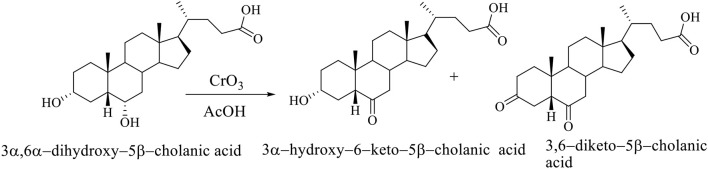
The selective oxidation of the C_6_ OH group of 3α,6α-dihydroxy−5β-cholanic acid (hiodeoxycholic acid), where are obtained 3α-hydroxy–6–keto–5β-cholanic acid and 3,6–diketo–5β-cholanic acid (Kuhajda et al., 2007).

The selective oxidation of C_6_ OH group of hiodeoxycholic acid (3α,6α-dihydroxy-5β-cholanic acid) is carried out using chromium (VI) oxide in acetic acid. The selective oxidation of the C_6_ OH group of the molecule of 3α,6α-dihydroxy–5β-cholanic acid (hyodeoxycholic acid) gives the 3α-hydroxy–6-keto–5β-cholanic acid and 3,6-diketo–5β-cholanic acid (Scheme 4; Kuhajda et al., 2007).

**Scheme 5:**
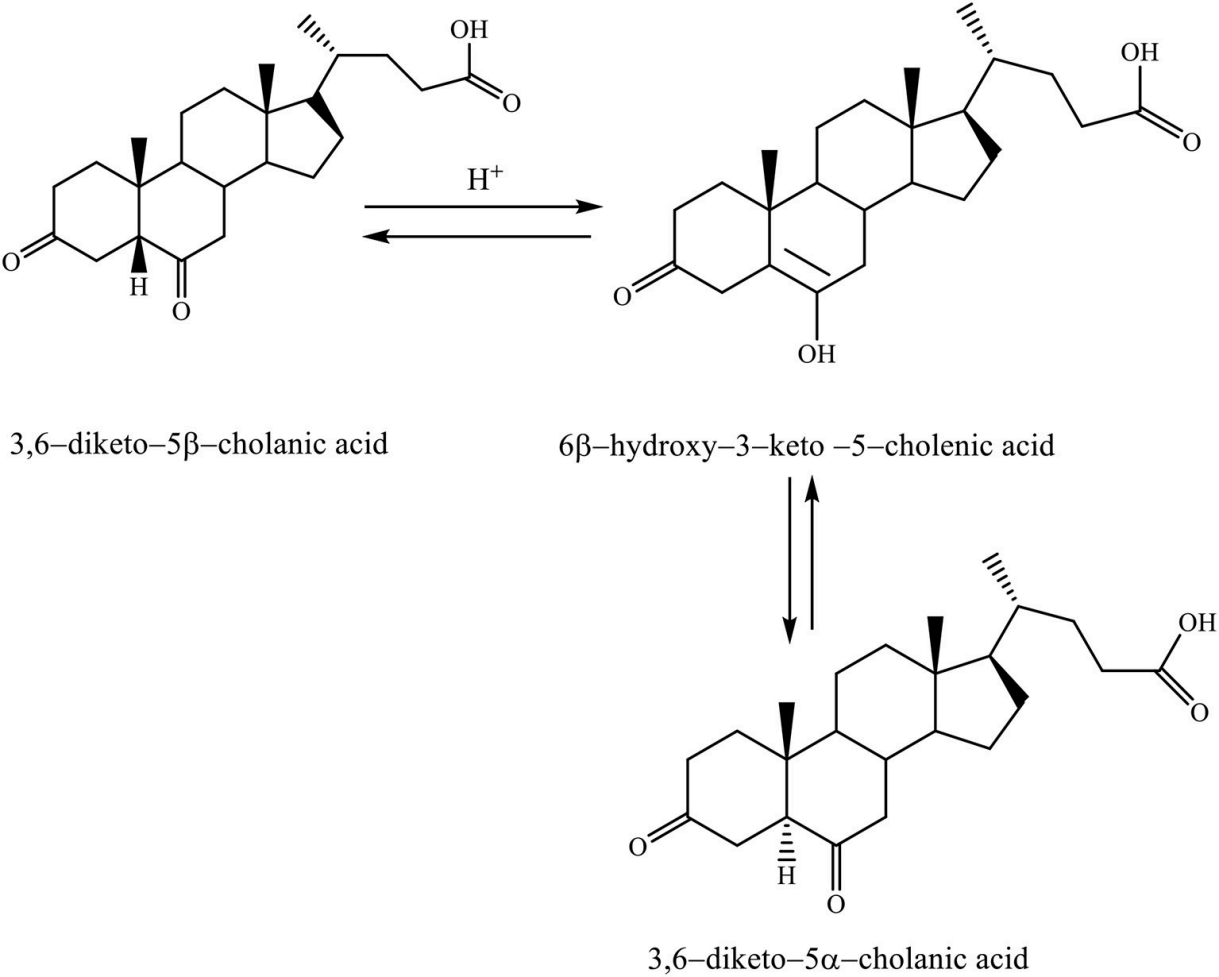
Heating reaction of 3,6–diketo−5β-cholanic acid in acetic acid with a catalytic amount of hydrochloric acid via the enol intermediate resulting in isomerization of 5β-hydrogen into 5α-hydrogen (Kuhajda et al., 2007).

When the 3,6–diketo−5β-cholanic acid is heated in acetic acid with a catalytic amount of hydrochloric acid, then it leads to the isomerization of 5β-hydrogen into 5α-hydrogen. The reaction is carried out via the enol intermediate (6β-hydroxy−3–keto−5–cholenic acid), thereby, forming the 3,6–diketo−5α-cholanic acid (Scheme 5; Kuhajda et al., 2007).

This transformation is possible for each steroid compound, which has a keto group in the C_6_ position. Isomerization takes place by the keto–enol tautomeric mechanism (Kuhajda et al., 2007).

**Scheme 6:**
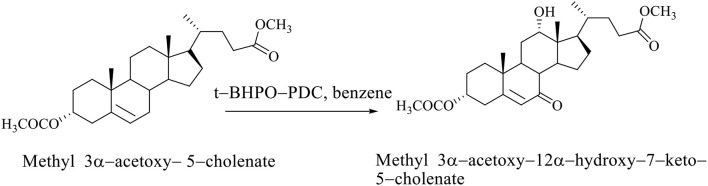
Oxidation reaction of methyl 3α-acetoxy−5–cholenate with tert–butylhydroxyperoxide whereby the methyl 3α-acetoxy−12α-hydroxy−7–keto−5–cholenate is obtained (Kuhajda et al., 2007).

If the steroid skeleton of BAs has a double bond, then allyl oxidation is possible, that is, the regioselective introduction of the oxo group into the allyl position. In the oxidation reaction of methyl 3α-acetoxy−5–cholenate with tert-butylhydroxyperoxide pyridinium dichromate (*t*-BHPO-PDC), the methyl 3α-acetoxy−12α-hydroxy−7–keto−5–cholenate (Scheme 6; Kuhajda et al., 2007) is obtained.

**Scheme 7:**
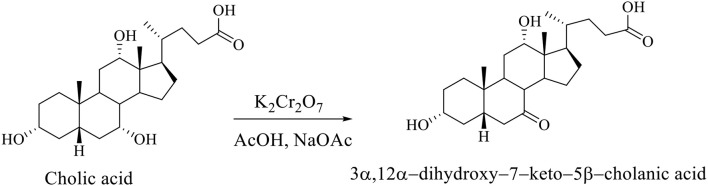
Regioselective oxidation of CA with an aqueous solution of potassium dichromate in acetic acid in presence of sodium acetate gives the 3α,12α-dihydroxy−7–keto−5β-cholanic acid (Kuhajda et al., 2007).

Regioselective oxidation of CA is carried out with an aqueous solution of potassium dichromate in acetic acid in the presence of sodium acetate, whereby the 3α,12α-dihydroxy−7–keto−5β-cholanic acid is formed (Scheme 7; Kuhajda et al., 2007).

**Scheme 8:**
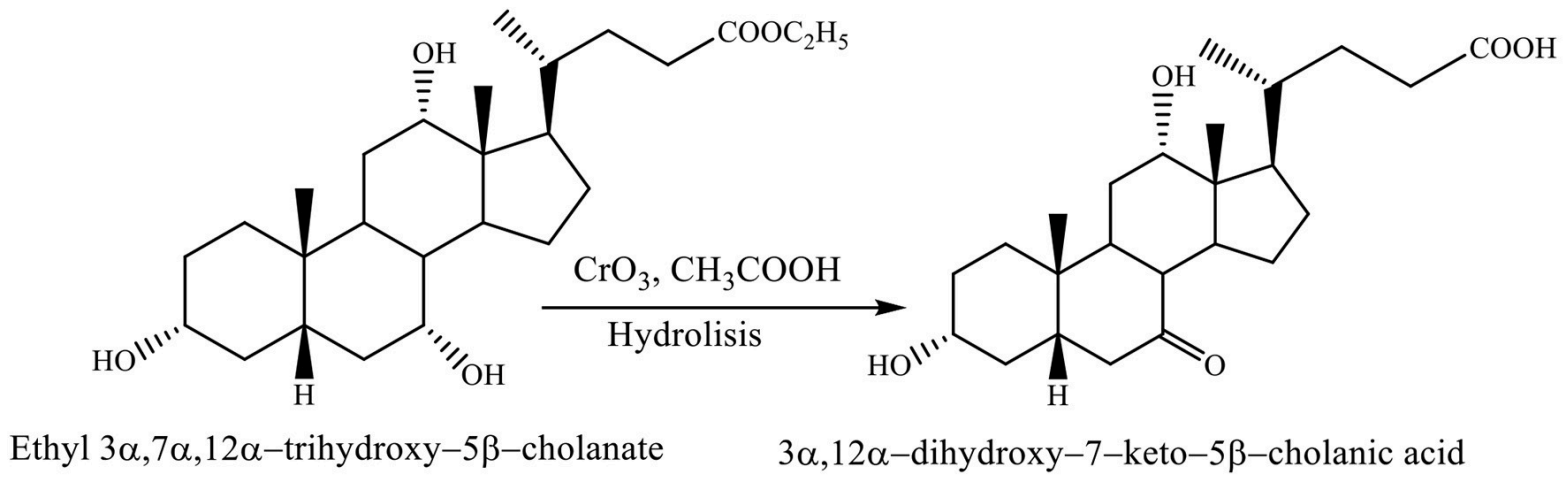
Both reactions, selective oxidation of ethyl 3α,7α,12α-trihydroxy−5β-cholanate with chromium trioxide in acetic acid and reaction of hydrolysis give the corresponding product (3α,12α-dihydroxy−7–keto−5β-cholanic acid) (Kuhajda et al., 2007).

Both reactions, selective oxidation of ethyl 3α,7α,12α-trihydroxy−5β-cholanate with chromium trioxide in acetic acid at −7 to 0°C and reaction of hydrolysis involve forming the 3α,12α-dihydroxy−7–keto−5β-cholanic acid (Scheme 8; Kuhajda et al., 2007).

**Scheme 9:**
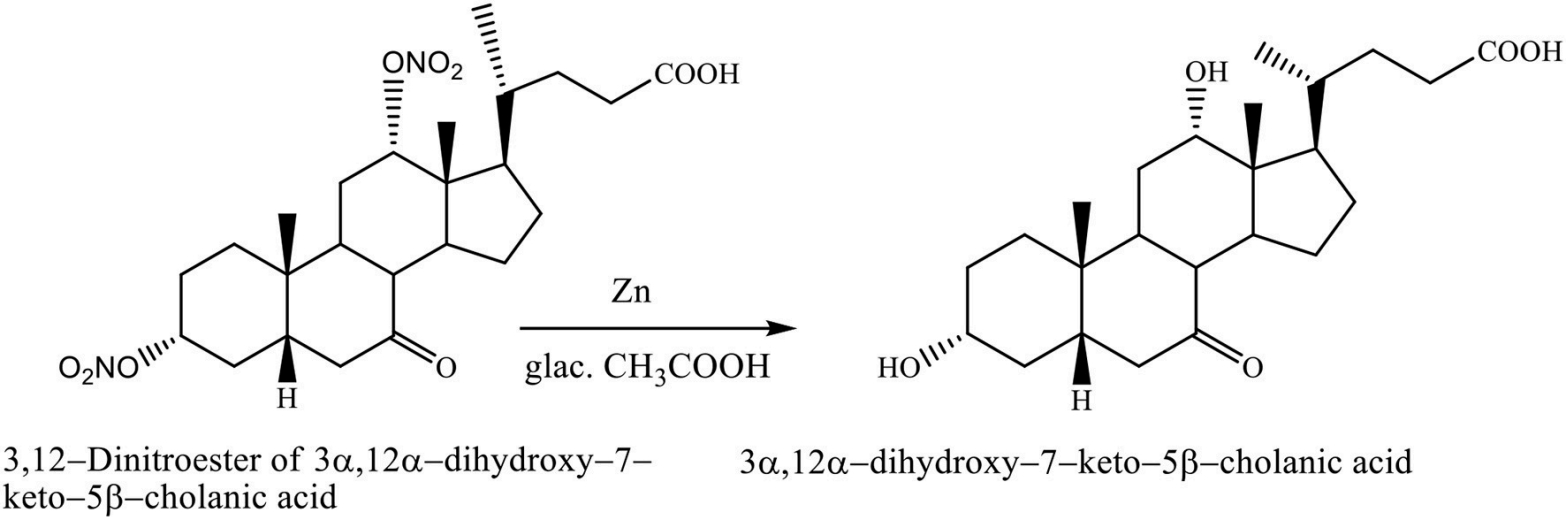
Transformation reaction of 3,12–Dinitroester of 3α,12α-dihydroxy−7–keto−5β-cholanic acid to the 3α,12α-dihydroxy−7–keto−5β-cholanic acid (Kuhajda et al., 2007).

The nitrate groups of 3,12–dinitroester of 3α,12α-dihydroxy−7–keto−5β-cholanic acid were removed with zinc in glacial acetic acid, yielding the 3α,12α-dihydroxy−7–keto−5β-cholanic acid (Scheme 9; Kuhajda et al., 2007).

**Scheme 10:**
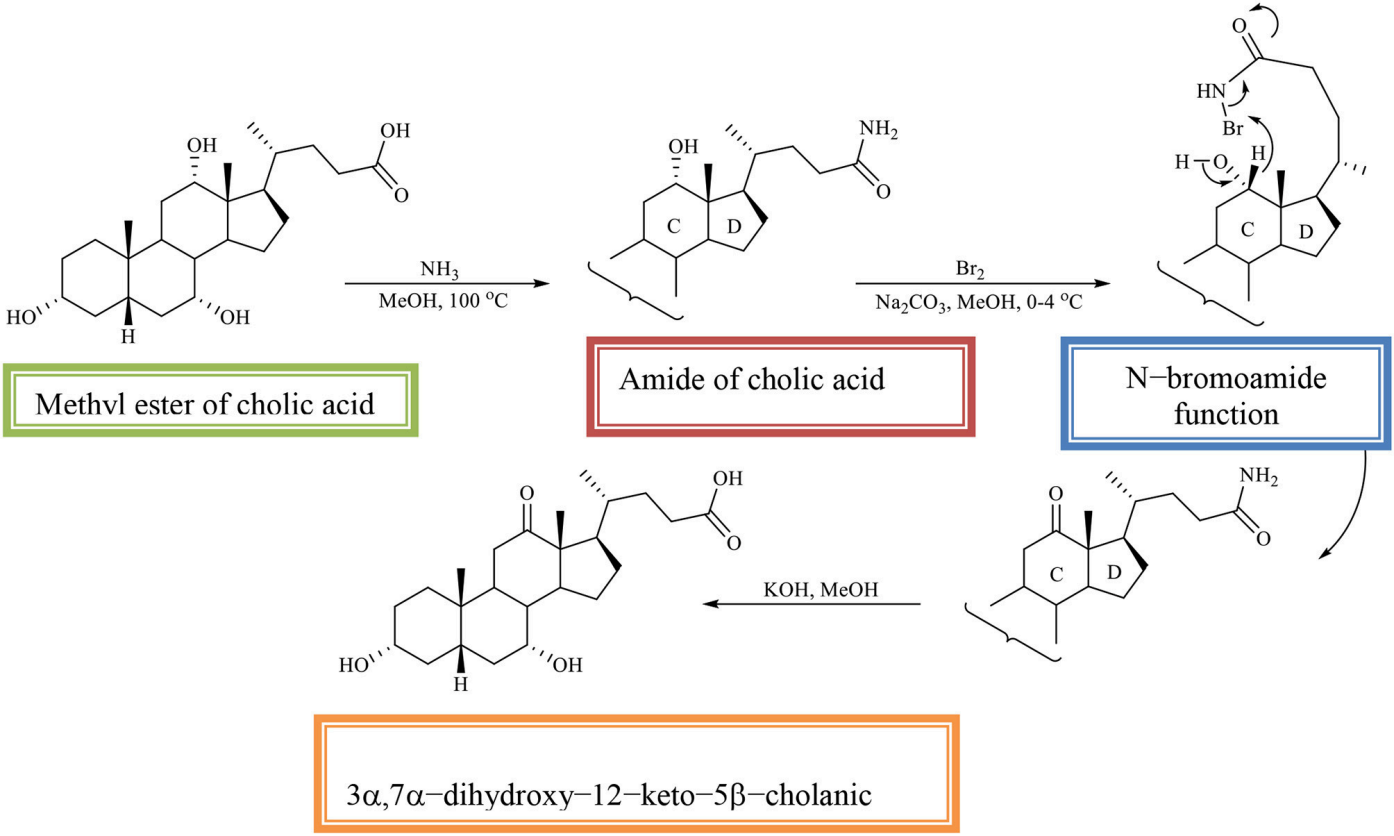
Regioselective oxidation of C_12_ OH group of the methyl ester of cholic acid to obtaines the 3α,7α-dihydroxy−12–keto−5β-cholanic acid (Kuhajda et al., 2007).

The methyl ester of CA is converted into the amide of CA, which is then oxidized with an equivalent amount of bromine in alkaline methanol. The regioselectivity of the reaction is determined by the *N*–bromoamide function from the side chain. In the reaction, the 3α, 7α-dihydroxy−12–keto−5β-cholic acid is obtained (Scheme 10; Kuhajda et al., 2007).

We have also considered the synthesis of 3α,7α, 16α-trihydroxy-5β-cholan-24-oic acid (avicholic acid) and natural BA first isolated from avian species (Shoebill stork and herons) and its derivatives 5−7 as TGR5 ligands (Mukhopadhyay and Maitra, 2004b). 6alfa-Ethyl-Avicholic acid sodium salt, 3alfa,7alfa,16beta-trihydroxy-5beta-cholan-24-oic acid sodium salt and 3alfa,7alfa,16beta-trihydroxy-6alfa-ethyl-5beta-cholan-24-oic acid sodium salt.

Development of ligands for BA-activated receptors, starts from cholic acid (CA) as a lead compound and INT-777 as a potent and selective TGR5 agonist *in vivo*. INT-777 is able to stimulate type 2 iodothyronine deiodinase (D_2_) activity in brown adipose tissue (BAT) and muscle, as well as induce the release of glucagon-like protein 1 (GLP-1) in enteroendocrine cells.

Avicholic acid derivatives were prepared according to the synthetic approach reported previously by Mukhopadhyay. Chenodeoxycholic acid and 6α–ethyl chenodeoxycholic acid were treated with *p*-toluensulfonic acid (*p*TSA) and MeOH at room temperature to get the corresponding methyl ester analogs. They were selectively protected at the C_3_ position by reaction with acetic anhydride in the presence of NaHCO_3_ in refluxing THF.

**Scheme 11:**
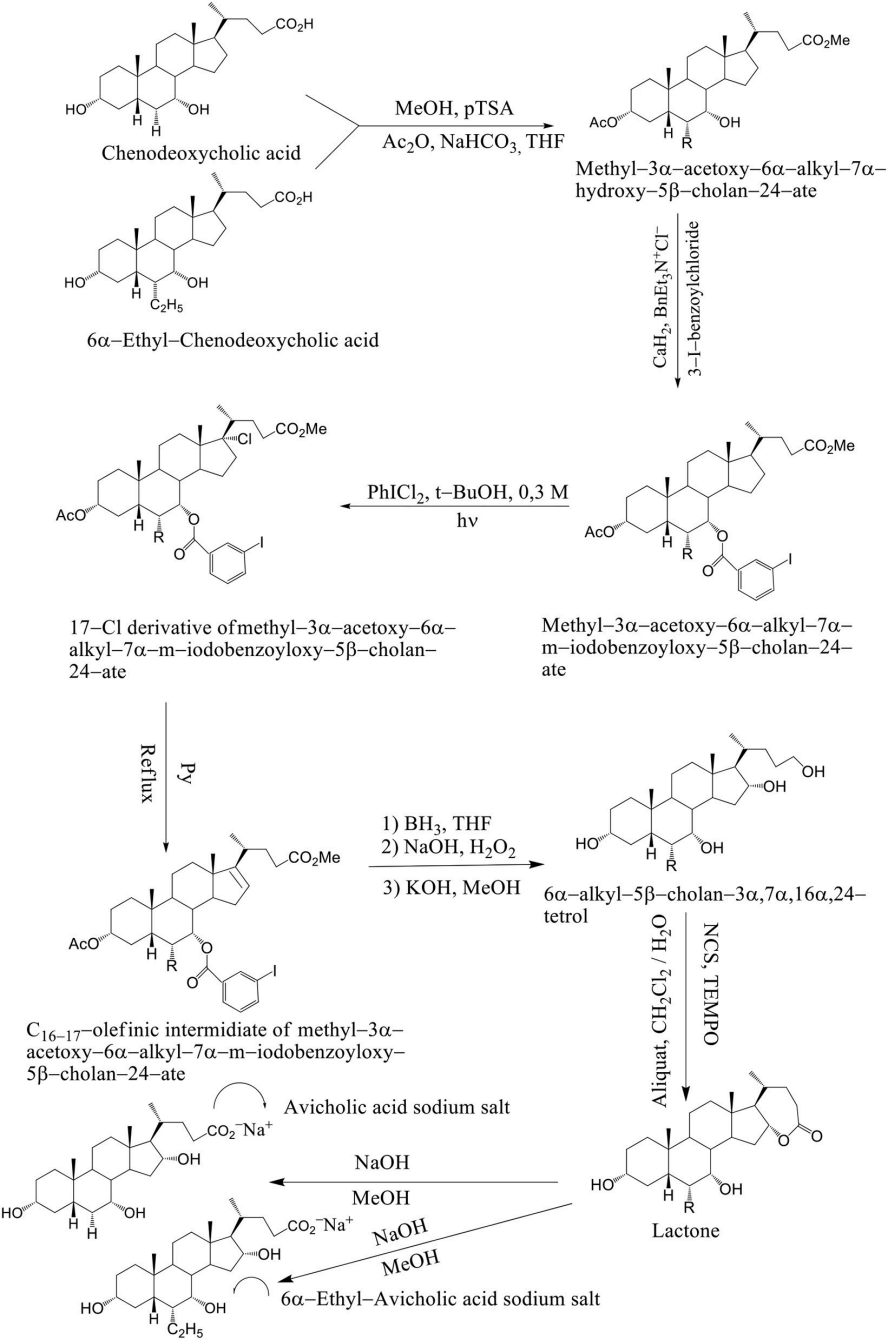
Synthesis of Avicholic Acid Derivatives. Reagents and conditions: (i) (1) MeOH, *p*TSA; (2) Ac_2_O, NaHCO_3_, THF. (ii) 3-I-benzoylchloride, CaH_2_, BnEt_3_N^+^Cl^−^. (iii) PhICl_2_, *t*-BuOH 0.3 M, hυ. (iv) Py, reflux. (v) (1) BH_3_·THF; (2) NaOH, H_2_O_2_; (3) KOH, MeOH. (vi) NCS, TEMPO, Aliquat, CH_2_Cl_2_/H_2_O. (vii) NaOH, MeOH.

The methyl 3α-acetoxy–6α–alkyl–7α–hydroxy–5β–cholan–24–ate was functionalized at the C_7_ position with a 3-iodo-benzoyl moiety by reaction with 3-iodo-benzoylchloride in the presence of CaH_2_ and BnEt_3_N^+^Cl^−^ in toluene to afford methyl–3α–acetoxy–6α–alkyl–7α–m–iodobenzoyloxy–5β–cholan–24–ate in good yield (Mukhopadhyay and Maitra, 2004b). Breslow's photolysis of PhICl_2_ in the presence of methyl 3α–acetoxy–6α–alkyl–7α–m–iodobenzoyloxy–5β–cholan–24–ate was achieved by 0.3 mM tBuOH / CH_2_Cl_2_ solution to yield the 17Cl–derivative of methyl–3α–acetoxy–6α–alkyl–7α–m–iodobenzoyloxy–5β–cholan–24–ate in quantitative yield. C_16−17_ olefinic intermediate was obtained by refluxing 17Cl–derivative of methyl–3α–acetoxy–6α–alkyl–7α–m–iodobenzoyloxy–5β–cholan–24–ate in pyridine. The 6α–alkyl–5β–cholan–3α,7α,16α,24–tetrol was obtained by hydroboration-oxidation of C_16−17_ olefinic intermediate by borane-THF complex and alkaline hydrogen peroxide, followed by base hydrolysis (NaOH/MeOH). It is carried out a selective oxidation of the C_24_ position was achieved by using a (2,2,6,6–tetramethylpiperidin–1–yl) oxyl (TEMPO) in CH_2_Cl_2_ / H_2_O and Aliquat as a phase transfer catalyst to afford the lactone. Alkaline hydrolysis (NaOH / MeOH) was afforded avicholic acid sodium salt and 6α-ethyl-avicholic acid sodium salt (Mukhopadhyay and Maitra, 2004b) (Scheme 11).

**Scheme 12:**
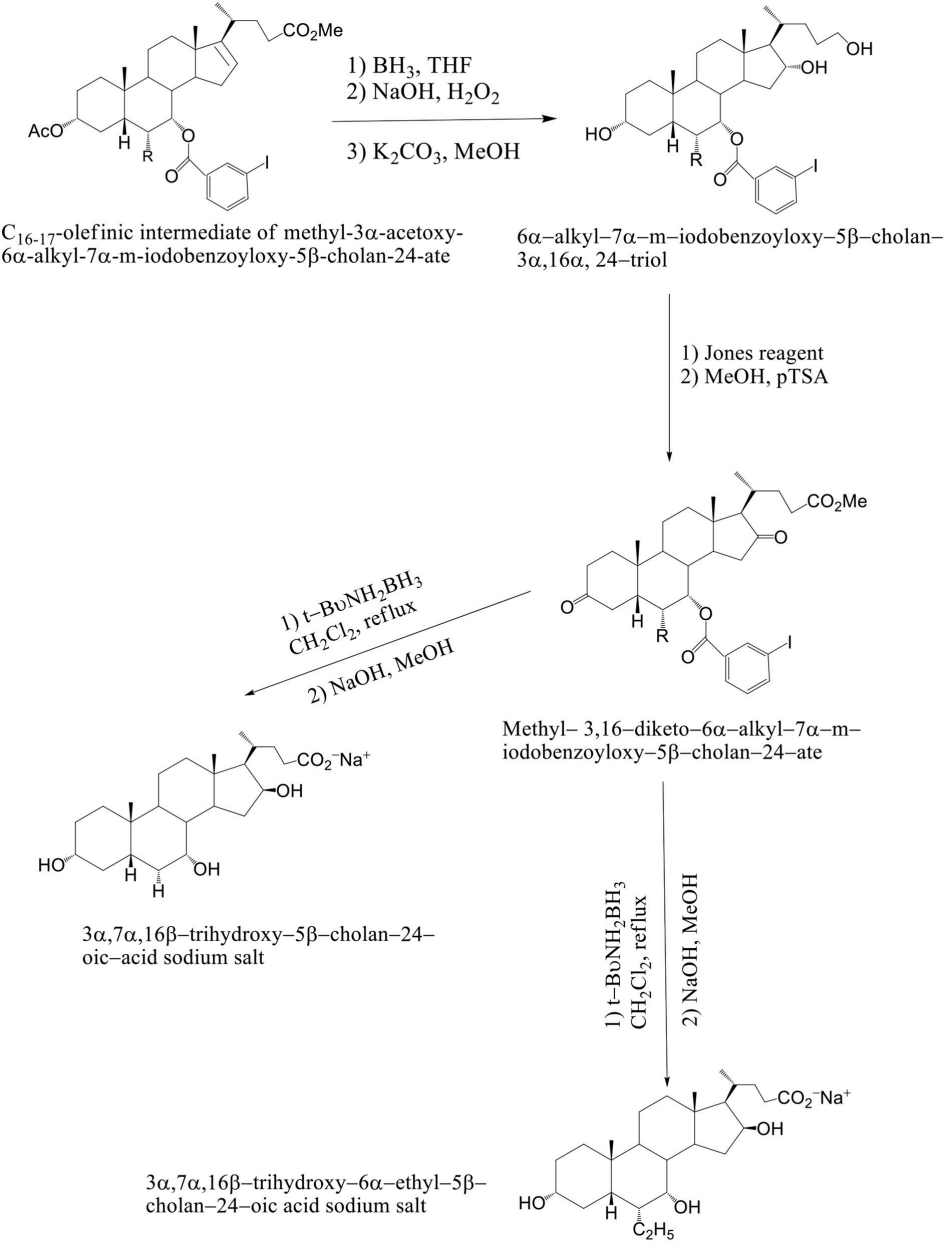
Synthesis of 16-epi-Avicholic Acid derivatives. Reagents and conditions: [(i) (1) BH_3_·THF; (2) NaOH, H_2_O_2_; (3) K_2_CO_3_, MeOH. (ii) (1) Jones reagent; (2) MeOH, *p*TSA. (iii) (1) *t*-BuNH_2_BH_3_, CH_2_Cl_2_, reflux; (2) NaOH, MeOH].

The solution of K_2_CO_3_ in CH_3_OH at room temperature is used, when the C_16_–C_17_ olefinic derivative of methyl–3α–acetoxy–6α-alkyl–7α–m–iodobenzoyloxy–5β–cholan–24–ate was submitted to hydroboration–oxidation reaction and selectively deprotected at C_3_ position. The 6α–alkyl–7α-*m*-iodobenzoyloxy-5β-cholan-3α,16α,24–triol was reacted with Jones reagent and esterified at the terminal carboxylic group by treatment with *p*TSA in MeOH at room temperature to give methyl 3,16–diketo–6α-alkyl–7α–m–iodobenzoyloxy –5β–cholan–24–ate in nearly quantitative yield. By reduction of the keto groups with tert–butylamine–borane complex in CH_2_Cl_2_ at reflux, followed by hydrolysis with NaOH in CH_3_OH and purification by medium pressure liquid chromatography are formed 3α,7α,16β-trihydroxy-5β-cholan-24-oic acid sodium salt and 3α,7α,16β-trihydroxy-6α-ethyl-5β-cholan-24-oic acid sodium salt. Compounds 4–7 were unstable in the free acid form, spontaneously reacting to give the corresponding lactone derivatives (Mukhopadhyay and Maitra, 2004b) (Scheme 12). Avicholic acid sodium salt, 6alfa-Ethyl-Avicholic acid sodium salt, 3alfa,7alfa,16beta-trihydroxy-5beta-cholan-24-oic acid sodium salt and 3alfa, 7alfa,16beta-trihydroxy-6alfa-ethyl-5beta-cholan-24-oic acid sodium salt.

The (3α,12α,16β-trihydroxy-5β-cholan-24-oic acid, EPCA) is an epimer of pythocholic acid at snakes (3α,12α,16α-trihydroxy-5β-cholan-24-oic acid, PCA) and involves a series of simple and selective chemical transformations of CA. The CMC of 16-epi-pythocholate in aqueous media was determined using pyrene as a fluorescent probe. *In vitro* cholesterol solubilization ability was evaluated using anhydrous cholesterol, and results were compared with those of other natural di- and trihydroxy BAs. The 16-epi-pythocholic acid (16β-hydroxy-deoxycholic acid) behaves similar to CA and avicholic acid (3α,7α,16α-trihydroxy-5β-cholan-24-oic acid, ACA) in its aggregation behavior and cholesterol dissolution properties (Nonappa and Maitra, 2010). Pythocholic acid formed a methyl ester, which was oxidized by chromic oxide to the triketone methyl dehydropythocholate. The obtained compound was reduced by the Wolff–Kishner method to cholanoic acid (Nonappa and Uday, 2007).

Pythocholic acid contains three secondary hydroxyl groups attached to the cholanic acid nucleus.

**Scheme 13:**
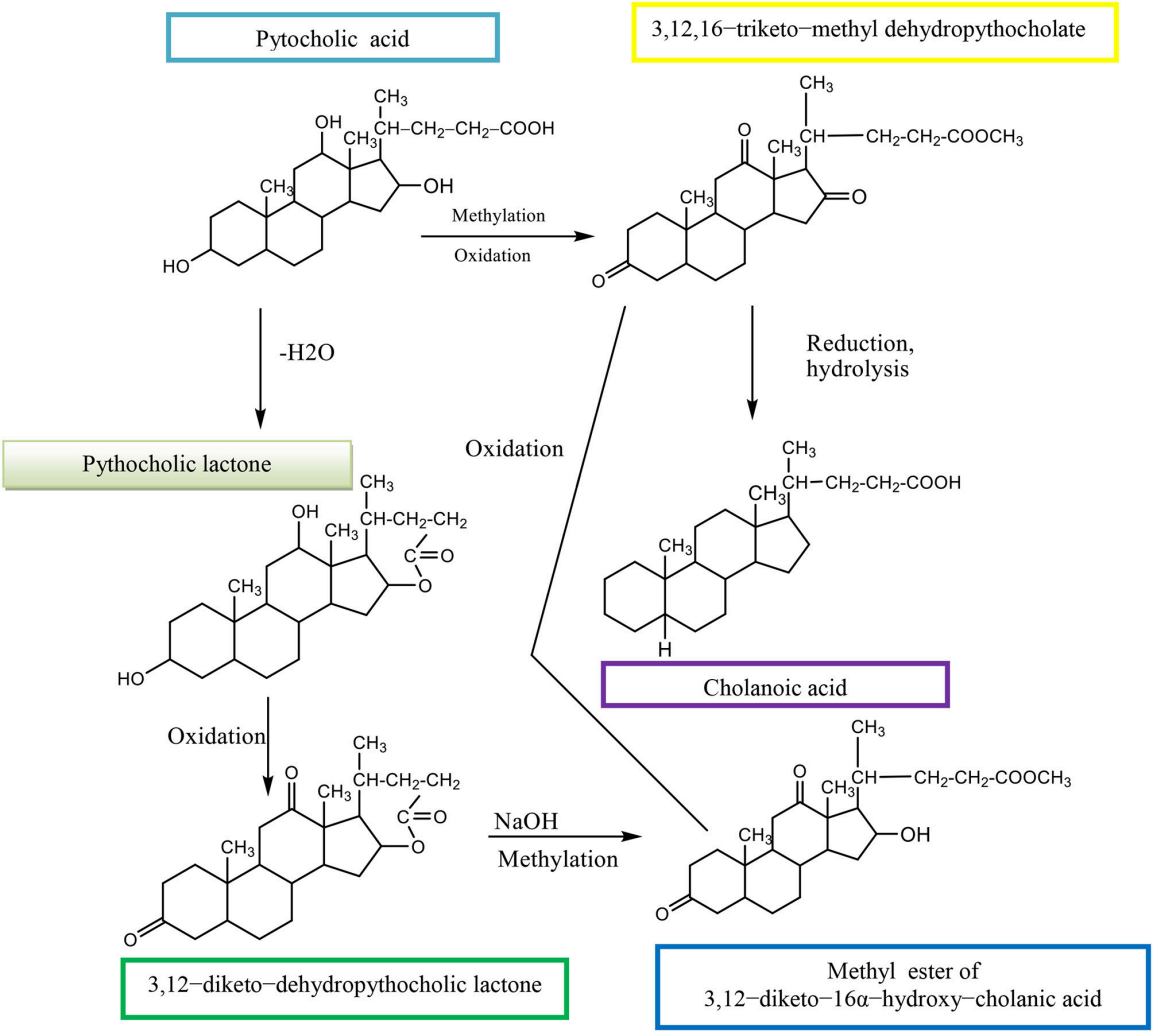
Synthesis of methyl ester of 3,12–diketo−16α-hydroxy–cholanic acid from the pythocholic acid.

Pythocholic lactone formed a diketone, dehydropythocholic lactone, by chromic oxidation. Then, the dehydropythocholic lactone reacts with NaOH and in methylation reaction occurs the methyl ester of 3,12–diketo−16α-hydroxy–cholanic acid (Scheme 13; Nonappa and Uday, 2007).

## The role of membrane transport proteins of BAs in the distribution of drugs

Many metabolic enzymes and transport proteins bind to corresponding substrates, such as different drugs. They are responsible for the metabolism and transport of BAs through cell membranes in both directions. It is important to note that nuclear receptors and other ligand–dependent transcription factors have the role of sensor that detects the presence of drugs or BAs, and regulates the expression of metabolic enzymes and transport proteins in order to maintain homeostasis (Staudinger et al., 2013). Molecular mechanisms regulate the inducible expression of the gene by drugs and BAs, where key mediators in these processes are nuclear subfamily 1 receptors [pregnane X receptor (PXR), constitutive androstane receptor (CAR), FXR, and vitamin D receptor (VDR)]. Membrane transport proteins regulate the transport of substrates and, for this reason, they represent the essential regulators of absorption, distribution, metabolism, excretion, and toxicity (ADMET) of any substrate in body, both natural ligands and metabolites and medicaments (Kramer, 2011). Also, many xenobiotics are involved in the transport roads common to physiological intermediates. Many physiological transporters are not monospecific, but have a wide range of specific substrate. Interactions of substrate (endogenous metabolite or drug / xenobiotics) with transport proteins have a very significant effect on both the efficacy and safety profile of the drug. The most important bile acid transport proteins with a wide range of substrate specificities of great importance for drug action and disposition are the influx transporters from the group of organic anion transporters that belong to the solute carrier (SLCO) gene family as well as an efflux ABC transporter protein. For some transporter proteins from the above–metioned groups, BAs represent physiological substrates with proven effects on absorption, distribution, metabolism, excretion, and toxicity features of many medicaments (substrates for said membrane transporters) (Kramer, 2011). Transport proteins for the substrate acquisition have developed evolutionarily for the purpose of facilitating not only the takeover of cell nutrients and vitamins, but also resorption of endogenous products such as glucose and other carbohydrates, amino acids, and small peptide or BAs. Many of these transporters use an electrochemical gradient of ions such as Na^+^ for transport versus a concentration gradient (Staudinger et al., 2013).

Among the transport proteins for the transmission of BAs is OATP1A2, responsible for transporting various endogenous substrates (BAs, steroid conjugates, prostaglandins, thyroid hormones T_3_, T_4_, and rT_3_), and exogenous substrates (rocuronium, fexofenadine, MRI contrast, etc.) from the intestinal lumen and port of circulation. The expression of this transporter is controlled by PXR and VDR (Claro da Silva et al., 2013). The transport protein OATP1B1 is used to carry endogenous substrates (cholate, thyroxine, bilirubin, leukotrien, C_4_ and E_4_, estradiol 17β-glucuronide, etc) and clinically relevant drugs (statins, rifapmicin, enalapril, methotrexate, olmesatran) (Claro da Silva et al., 2013). This transporter is located on the basolateral membrane of the hepatocytes, and it is transcriptionally controlled by the PXR and FXR receptors. The OATP1B3 (SLCO1B3) is a hepatic–specific transporter from the OATP family, which mediates transfer of Na^+^–independent xenobiotics (docetaxel, enalapril, erythromycin, fexofenadine, fluvastatin, methotrexate, rifampin, and pacitaxel) and plays a key role in transmission of BAs and bilirubin. The OATP1B1 and OATP1B3 are transporters in the embryonal kidney cells, HEK293, and they are responsible for transport of CA, CDCA, and DCA (Claro da Silva et al., 2013). It has been proven that conjugated glycin and taurine derivatives of these BAs (glycochenodeoxycholic acid, taurochenodeoxycholic acid, glycodeoxycholic acid, taurodeoxycholic acid, glycolithocholic acid, and taurolithocholic acid) are natural substrates for OATP1B1 and OATP1B3. At physiological pH = 7.4, unconjugated BAs pass through the cell membrane by passive diffusion, while the transmission of conjugated BAs to be mediated by transporters is necessary. Chenodeoxycholic acid is the most potential endogenous FXR agonist, which induces the promoter activity of transport protein in HepG2 and Huh 7 cell lines. Rifampicin, a potent PXR activator, reduces the expression of the transport protein to the basolateral hepatocyte membrane. Transporter NTCP (SLC10A1) localized to the basolateral hepatocyte membrane and transporter ASBT (SLC10A2) localized to the apical enterocyte membrane are Na^+^–dependent transporters, whose ligands are conjugated and unconjugated BAs. Both transporters are under the negative transcriptional control of FXR (Claro da Silva et al., 2013).

### Application of BAs in the development of new drug formulation

The BAs are used as drug delivery systems. They can play a role of drug carrier in the form of mixed micelles, liposomes stabilized by BAs (bilosomes), and chemical conjugates with different drugs (Faustino et al., 2016). Organotropicity of BAs and affinity for protein transport systems within the enterohepatic system have been used for the selective therapeutic targeting of liver tissue to improve intestinal absorption and the metabolic stability of drug. The BAs can represent carrier linkers of various structures, lengths, charges, functions, and stereochemistry. The BAs allow addition of different drugs to one of the functional groups, including the hydroxyl groups at positions C_3_, C_7_, C_12_ or the side chain carboxyl group at C_24_ position. These conjugates get involved in the interactions with bile acid transporters (Stojančević et al., 2013). The BAs may alter the pharmacokinetic and pharmacodynamic properties of the medicinal substance, which they carry. In accordance with the predictions of the quantitative relationship of structure and activity, QSAR, on transporter models, the most effective molecular recognition by the membrane transporter of hepatocyte and ileal enterocyte is achieved linking the drug at the C_3_ position of BAs (Faustino et al., 2016). The hydroxymethylglutaryl (HMG)–CoA reductase conjugate associated with an amide linkage to the C_3_ position of the CA derivative (S−3554) with a free C_24_ carboxyl group has led to a specific inhibition of cholesterol biosynthesis in hepatocytes. The corresponding taurine conjugate released through in the portal vein is transported unchanged through hepatocytes and excreted into the bile without the intracellular drug release and inhibition of HMG–CoA reductase. The intracellular transfer of the drug and the availability of organelles can be coordinated by the conjugation status of bile acid side chain. Chlorambucil–taurocholate conjugate, S2776, exhibited all the pharmacokinetics properties of the bile acid conjugate, including specific interactions with the ileal transporter, ileal bile acid-binding protein (IBAB–P) (Stojančević et al., 2013).

Uptake transporters may facilitate drug transfer from blood to liver for further processing by metabolizing enzymes and/or excretory transporters. There is a change in the pathway of drug secretion from the renal to the biliary pathway and in transcellular transport of drug from the blood to the bile in competition with natural BAs. NTCP is the main transporter accounting for the liver uptake of conjugated bile acids (e.g., taurocholate, tauroursodeoxycholate and taurochenodeoxycolate), but it is also able to transport, although with less efficiency, non conjugated bile acids. OATPs are membrane influx transporters that regulate cellular uptake of a number of endogenous compounds and clinically important drugs. The ASBT transport system has the ability to recognize the drugs and peptides as substrates of bile acid conjugates, which potentially can result in the increase of the intestinal resorption of the drug. It is possible to add the peptide to the C_24_ position of the bile acid side chain, but the resorption of such conjugates in the ileum is small, since the negative charge of the bile acid side chain is necessary for molecular recognition by the transporter (Faustino et al., 2016).

### The BAs as therapeutic agents (drugs)

The BAs and their derivatives may possess therapeutic effects in the treatment of the human type of immunodeficiency virus 1 (HIV 1). Difficulties in the delivery of BAs in the blood explain why BAs are not fully used as therapeutic agents against wrapped viruses. Taurolithocholic acid 3–sulfate is very effective against *Herpes simplex* virus 1 and 2, HIV, *Neisseria gonorrhoeae* and *Chlamydia trachomatis* with low and non citotoxicity to human cervical epithelial cells (Mikov et al., 2007). Ursodeoxycholic acid plays a role in improving hepatic histology and reduces serum bilirubin level as an important analytic marker in primary biliary cirrhosis (PBC). Long–term liver survival after transplantation was established in patients who received 13–15 mg / kg / day of UDCA for a period of four years. The UDCA acid was used in patients with primary sclerosing cholangitis in several doses over a period of one year (Mikov et al., 2007). Sclerosing cholangitis is characterized by chronic inflammation of intrahepatic and extrahepatic gallbladder, leading to fibrosis and possible damage of bile ducts. In addition to cholecystectomy, and laparoscopic cholecystectomy, BAs are used for the removal of gallstones. Chenodeoxycholic acid reduces the activity of HMG–CoA reductase, an enzyme that limits the rate of cholesterol formation. Size of stones in the bile is an important factor in the treatment with BAs. In patients, who were treated with UDCA, it has been found that gallstones were completely destroyed after two years of treatment. The UDCA Ursodeoxycholic acid is effective in the treatment of choledocholithiasis and hepatolithiasis associated with Caroli syndrome. The UDCA and CDCA have shown antiproliferative effect and they are able to induce apoptosis in carcinogenic cells (Mikov et al., 2007). Obeticholic acid (OCA) is used for the treatment of PBC. Primary biliary cholangitis, previously known as primary biliary cirrhosis, is a chronic cholestatic liver disease with an autoimmune basis, affecting mostly middle-aged women (Momah and Lindor, 2014). In 2017, the FDA approved FXR agonist and, currently, numerous FXR agonists are under clinical trials for nonalcoholic fatty liver disease/nonalcoholic steatohepatitis (NAFLD/NASH) (Le et al., 2012). The NAFLD refers to a spectrum ranging from noninflammatory isolated steatosis to NASH, which is characterized by steatosis, necroinflammatory changes, and varying degrees of liver fibrosis (Le et al., 2012). The OCA is a derivative of CDCA, the primary human BA, and it is the natural ligand for FXR. By activating FXR in the ileum, OCA decreases BA reabsorption through downregulation of the apical sodium-dependent BA transporter and increased expression of fibroblast growth factor (FGF) 19, which, in the liver, also decreases BA synthesis through CYP7A1. The OCA may have antifibrotic properties and the potential to improve portal hypertension (Hirschfield et al., 2009). Ligand-activated nuclear receptors control key steps in lipid metabolism as well as inflammation and fibrogenesis and thus, are potentially crucial players in NAFLD/NASH pathogenesis (Le et al., 2012). Such a receptor is the FXR. The FXR is a key regulator of hepatic lipid metabolism. The role of FXR has been emphasized on by the development of hepatosteatosis and hyperlipidemia in FXR^−/−^ mice (Ali et al., 2015). Farnesoid X receptors are considered nuclear hormone receptors, due to their actions in the nucleus of the hepatic and intestinal cells. In the enterocytes, the activity of Farnesoid X receptor is reflected in the regulation of bile acid synthesis by releasing FGF−19 into the portal circulation (Ali et al., 2015). Farnesoid X receptors are involved in the modulation of hepatic inflammation, fibrosis, metabolic pathways, and regeneration. The FXRs facilitate bile salt secretion through the downregulation of the bile salt export pump (Ali et al., 2015). The FXR agonists are classified into important regulators of bile acid metabolism pathways, which are involved in regulation of the production and flow of BAs in the liver. The OCA is a first class agonist, which selectively binds to FXR. The OCD increases insulin sensitivity and reduces markers of liver inflammation and fibrosis. The OCA has significant beneficial effects on NASH related liver health (Hirschfield et al., 2015). It is also indicated for the treatment of PBC in combination with UDCA in adults with an “inadequate” response to UDCA or as monotherapy in adults unable to tolerate UDCA. The BA transporters modulators or BA sequestrants could have beneficial therapeutic effects in (NAFLD / NASH) (Trivedi et al., 2016; Arab et al., 2017). Treatment with OCA led to a significant reduction of liver fibrosis as compared with patients treated with placebo (Kim et al., 2000). The OCA has a role in the regulation of lipid, glucose, and energy homeostasis, and it is a potential target for the treatment of obesity and NAFLD. Also, intestine-specific FXR agonist, Fexaramine, has been shown to increase energy expenditure to reduce body weight gain in obese animal model (Neuschwander-Tetri et al., 2015).

Secondary BAs produced in the intestine (colon) by gut bacteria activate TGR5 signaling, which induces cAMP/PKA signaling to stimulate energy metabolism in BAT, relax and refill the gallbladder, and secrete GLP-1 from the intestinal endocrine L cells (Reich et al., 2016).

## Hypoglycemic effect of BAs and their application in treatment of diabetes mellitus

The BAs have the ability to act as permeation enhancers for antidiabetic drugs over the ileal mucosa and through the blood–brain barrier. They also showed potential health benefits in the treatment of diabetes by their endocrine, metabolic, energy, and other effects. The strongest hypoglycemic effect in type 1 diabetes (T1D) is exhibited by 3α,7α-dihydroxy−12–keto−5β-cholanic acid, while still better effects are achieved when applying the mentioned 12–keto derivative of CA in combination with hypoglycemic agent gliclazide or a composition of stevioside (Mikov et al., 2008). It has been proven that the best effects of glycemic control has been achieved in the case when rats with T1D were prebiotically pretreated and then using 3α,7α-dihydroxy−12–keto−5β-cholanic acid and gliclazide simultaneously (Mikov et al., 2008). It has been known that the transporter function is disrupted or suppressed in diabetes. Contrary to *in vivo* results, *in vitro* studies have produced the opposite results. It has been found that the 3α,7α-dihydroxy−12–keto−5β-cholanic acid acts as an inhibitor of the efflux transporter and the transfer of substances in the direction from mucosa to the serosa via the inhibition of MrP_3_ transporter. It is believed that this is a disagreement with the results of *in vivo* studies because in *in vivo* biotransformations (metabolic transformations), mono keto derivatives enhance *in vivo* absorption of gliclazide in ileum (Al-Salami et al., 2008).

In intravenous administration (independent of the use of gliclazide or probiotic pretreatment), the pharmacokinetic properties of 3α,7α-dihydroxy−12–keto−5β-cholanic acid remain unchanged, but they are significantly changed in case of oral administration. It has been proven that 12–ketocholic acid enhances the nasal absorption of insulin in rats (Al-Salami et al., 2008).

Gliclazide is used in the treatment of type 2 diabetes (T2D) to help stimulate the insulin production. It also has beneficial extrapancreatic effects, which makes it potentially useful in T1D. Some patients with T2D continue to use gliclazide even after their diabetes progresses to T1D, since it provides better glycemic control than insulin alone (Dutta et al., 2016).

The administration of gliclazide and 3α,7α-dihydroxy−12–keto−5β-cholanic acid allows the most significant reduction in blood glucose level in probiotic–pretreated diabetic rats (from 12.6 ± 2.0 to 10 ± 2.0 mmol / l, *p* < 0.01). Pretreatment with probiotics and subsequent oral administration of gliclazide + 12–ketocholic acid resulted in the greatest effect in the treatment of T1D, as well as in improved signs and symptoms in the animals. (Al-Salami et al., 2009)

The effect of oral 3α,7α-dihydroxy−12–keto−5β-cholanic acid was not significant in probiotic–pretreated diabetic rats that had lower blood glucose levels at the time of administration of the 12–keto derivative of CA, possibly due to an interaction in the gut (Al-Salami et al., 2009).

The combination of gliclazide + 3α,7α-dihydroxy−12–keto−5β-cholanic acid produced a greater effect in diabetic rats than 3α,7α-dihydroxy−12–keto−5β-cholanic acid alone (Mikov et al., 2008).

In the regulation of glycemic response in patients with T2D, an important effect of bile acid sequestrants in bariatric surgery has been demonstrated, which greatly affect the level of glucose and profile of BAs. Bile acid sequestrants have beneficial effects on the glycemic control and insulin sensitivity in patients with T2D and diabetic rodents. One bile acid sequestrant, colesevelam is an approved drug, used to treat diabetes (Brufau et al., 2010). Patients with T2D, who are treated with colesevelam, showed significant reduction in HbA1c and postprandial glucose levels, even when colesevelam was given in combination with other antidiabetic drugs. The healthy insulin–sensitive subjects remained unaffected by colesevelam treatment (Brufau et al., 2010). Patients with T2D and treated with colesevelam exhibited an increase in plasma triglyceride levels, which precludes colesevelam treatment in T2D subjects with hyperglyceridemia (Brufau et al., 2010).

Obesity and diabetic mice treated with colesevelam exhibited an improved glycemic response mediated by a double mechanism:

- The TGR5 mediated by GLP−1 secretion in L cells and
- intestinal expression of proglucagon

Bile acid sequestrants affect glucose absorption. The first dose of colesevelam with the standard meal had no effect on postprandial concentrations of glucose compared with baseline and placebo. Colesevelam did not appear to affect hepatic or peripheral insulin sensitivity as measured by the hyperinsulinemic–euglycemic clamp technique (Mari et al., 2011).

Neither acute nor chronic treatment with colesevelam seems to affect post–OGGT glucose concentrations (Li et al., 2012). Interventions by BAs and probiotics exert a direct and significantly positive effect on glycemic control and the progression of diabetic complications (Brufau et al., 2010).

**Figure 2:**
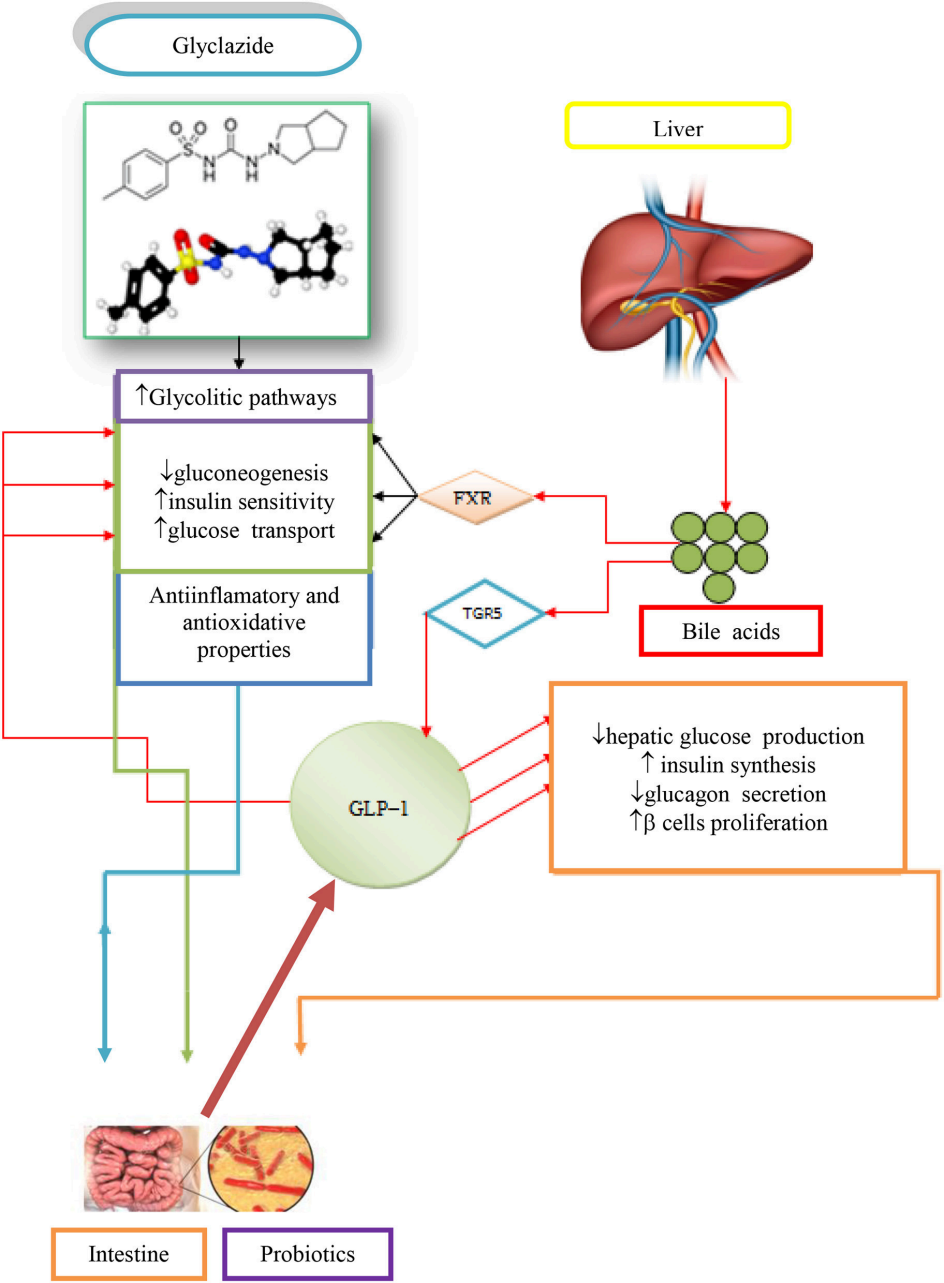
Potential synergistic effects due to concomitant administration of gliclazide, BAs and probiotics in treating T1D (Mikov et al., 2017).

The contribution of BAs, gliclazide, and probiotics in the regulation of glycemic responses in T1D is demonstrated in Figure 2 (Mikov et al., 2017).

Bile acid metabolism has been altered in patients with T2D (Zammitt and Frier, 2005). Modification of the bile acid pool leads to an improvement in glycemic control in such patients. The expression level of FXR in the liver has been reduced in diabetic animals. Activation of FXR through the small heterodimer partner (SHP) leads to a reduction in the expression of the gene for phosphoenolpyruvate carboxykinase (PEPCK) and glucose 6–phosphatase (G6Pase) involved in the process of gluconeogenesis (Zammitt and Frier, 2005).

## The role of BAs in digestion of nutrients

The BAs have important roles in lipid metabolism. They are essential for the formation of mixed micelles in the small intestine that facilitates solubilization, digestion, and absorption of dietary lipids and fat-soluble vitamins. The BAs function as nutrient–signaling hormones by activating specific nuclear receptors (FXR, PXR, vitamin D) and G-protein coupled receptors [TGR5, sphingosine-1 phosphate receptor 2 (S1PR2), muscarinic receptors].

The BAs and insulin appear to collaborate in regulating the metabolism of nutrients in the liver. They both activate the AKT and ERK1/2 signaling pathways. The disruption of these signaling pathways may increase the risk of fatty liver and NAFLD. Bile acid induction of the FXR-α target gene, small heterodimer partner (SHP), is highly dependent on the activation of the PKCζ, a branch of the insulin signaling pathway. The SHP is an important regulator of glucose and lipid metabolism in the liver. The BAs promote intestinal absorption of biliary and dietary lipids, prior to their return to the liver through the enterohepatic circulation or their excretion in the feces. The BAs are present in micellar concentrations and form mixed micelles with dietary lipids and their digestion products, such as monoacylglycerols and fatty acids (Russell, 2009).

The BAs also solubilize nonpolar lipids such as cholesterol and fat–soluble vitamin, increasing their water solubility and promoting their diffusion across the unstirred water layer for delivery to the intestinal epithelium. The BAs are powerful regulators of metabolism. Mice, when treated orally with CA, are protected from diet–induced obesity, hepatic lipid accumulation, and increased plasma triacylglycerol and glucose levels. The bile acid receptor (FXR) is involved in regulation of lipid and carbohydrate metabolism. Interruption of the enterohepatic recirculation of BAs, using bile acid sequestrants in patients with hypercholesterolemia or after ileal reaction, results in an increased level of triglycerides in the plasma. The FXR links the metabolism of BAs and triglycerides, such as via SHP protein, and reduces the expression of the transcription factor sterol regulatory element–binding protein 1c and its target genes for acetyl CoA synthetase, malic enzyme, and stearoyl–CoA desaturase 1, which are involved in biosynthesis of fatty acids and triglycerides. Activation of FXR stimulates β-oxidation of fatty acids and contributes to the reduction of lipid levels in the liver (Kalaany and Mangelsdorf, 2006).

Stimulation by FXR steroidal and nonsteroidal agonists leads to an increase in LDL levels and a decrease in HDL cholesterol levels. In humans, it has been confirmed that long–term administration of CDCA leads to a mild increase in serum LDL cholesterol concentrations. The effect of FXR on the expression of CYP 7A1 enzyme results in the inhibition of the conversion of cholesterol into BAs. Application of agonist FXR has a positive effect on the metabolism of glucose because it reduces insulin resistance and the concentration of glucose in the blood of animals (Kalaany and Mangelsdorf, 2006).

In the intestine, BAs are taken up into the enterocyte by the ASBT and SLC10A2, bound by the cytosolic ileal bile acid binding protein [IBABP; fatty acid binding protein 6 (FABP6)], and then exported across the basolateral membrane by the heteromeric, SLC51A; *O*STβ, SLC51B) (Dawson et al., 2010).

In the distal ileum, bile acid absorption from the lumen occurs via ASBT and bile acid efflux out of the cell via OSTα / OSTβ. The FXR target genes are SHP, FGF 15, IBABP, OSTα, and OSTβ, and secretion of FGF15 takes place into the portal blood.

**Figure 3:**
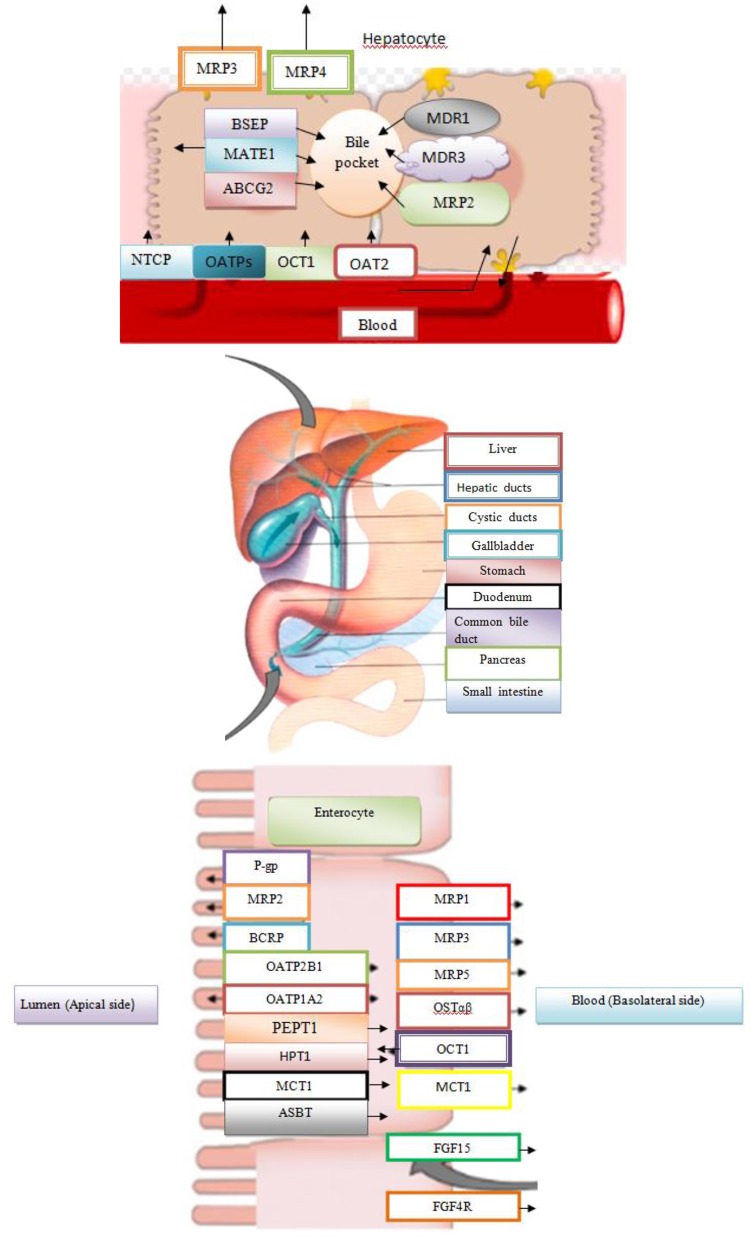
Bile Acid Metabolism in Liver and Intestine.

The BAs activate FXR in the liver inducing SHP, which inhibits the transcription of the *CYP7A1* and *CYP8B1* gene under physiological conditions. In the intestine, activated FXR induces FGF15/19, an intestinal hormone that induces FGFR4 on the hepatocytes and via cJUN inhibits CYP7A1. The FXR suppresses synthesis of BAs and also regulates its enterohepatic circulation. In the liver, FXR induces biliary bile acid excretion by BSEP and MRP2 transporters, which are located at the apical membrane of hepatocytes. The FXR induces hepatocyte basolateral transporters Ostα/β and MRP3/4, providing an alternative excretion route for BAs into the systemic circulation. In the ileum, FXR decreases reabsorption of conjugated BAs via ASBT, while inducing IBAP and Ostα/β promotes enterohepatic bile acid circulation. Face I (CYP3A4) and face II (SULT2A1 and UGT2B4) of bile acid detoxification are also positively regulated by FXR, rendering BAs more hydrophilic and less toxic (Figure 3; Stanimirov et al., 2012).

## Discussion

The amphiphilic nature of BAs is used to test the improvement of drug transport through biological membranes, allowing the design of new pharmaceutical formulations. Mainly, the mechanistic role of BAs is extended to the pleated regulatory functions, including cellular homeostasis, metabolic processes, regulation of cell proliferation, cell death, and the process of carcinogenesis. The BAs are recognized as paracrine and endocrine signaling molecules with the ability to activate different nuclear receptors such as the FXR, PXR, CAR, and VDR and membrane receptors–protein–coupled bile acid receptor (TGR5, GPBAR1) as well as various kinase signaling pathways that regulate the phosphorylation of histone and histone–regulatory proteins, thereby, affecting the regulation of gene expression involved in integrative metabolism. In addition to regulating the gene expression through nucleic receptors, the activation of the TGR5 receptor of bile acid exhibits genetic-independent effects. By activating the TGR5 receptor in enteroendocrine L cells, BAs improve glucose–induced insulin secretion and postprandial glycemia via glucagon like peptide−1 (GLP−1). Also, TGR5 is expressed in several regions of the central nervous system, where it has a role of neurosteroid receptor indicating that BAs have a far more important role than initially assumed. The alterations of bile acid homeostasis and bile acid–mediated signaling pathways contribute to the pathogenesis of hepato–biliary and intestinal diseases and disordes of metabolism of glucose and lipoproteins with the development of T2D, atherosclerosis with cardiovascular and cerebrovascular sequelae, NAFLD, inflammatory intestine disease, and neoplasms of gastrointestinal and hepatobiliary tract. The field of testing BAs has become extremely attractive to the scientific community in several research centers around the world with the aim of understanding the enigma of a complex network of signaling pathways mediated by BAs, improving the properties of existing drugs by developing new pharmaceutical formulations with BAs, and the development of new semisynthetics, which are analogs of BAs with the selective effect on nuclear and membrane receptors in order to prevent and treat various metabolic and nonmetabolic diseases. Three natural BAs are registered by regulatory agencies as medicines for human application. The UDCA has been used for about 30 years in the treatment of cholelithiasis and PBC. The Food and Drug Administration (FDA) has issued an authorization for the use of CA in the treatment of bile acid synthesis induced by enzymatic defect and as an additional treatment for peroxisomal disorders including Zellweger's cerebro-hepato-renal syndrome, as well as the injection of DCA in order to suppress submental fat tissue.

## Author contributions

All authors listed have made a substantial, direct and intellectual contribution to the work, and approved it for publication.

### Conflict of interest statement

The authors declare that the research was conducted in the absence of any commercial or financial relationships that could be construed as a potential conflict of interest.
